# Supplementary material for: Enhancing 1,3-Propanediol Productivity in the Non-Model Chassis Clostridium beijerinckii through Genetic Manipulation
Source: Microorganisms. 2023 Jul 22;11(7):1855. doi: 10.3390/microorganisms11071855 (PMC10383064; doi:10.3390/microorganisms11071855)
Supplement: Supplementary file 1 [file microorganisms-11-01855-s001.zip › microorganisms-2483047-supplementary.pdf]

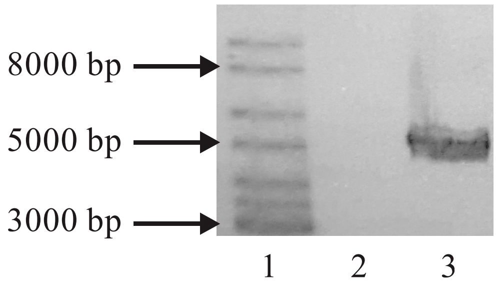

Figure S1. Gel electrophoresis representing the 1,3-PDO gene cluster amplification. Lane 1: GeneRuler DNA Ladder Mix (Thermo Fisher Scientific); Lane 2: PCR negative control, whose reaction was performed using water as replacement for DNA template. Lane 3: PCR product, whose reaction was performed using *C. beijerinckii* Br21 genomic DNA as template and dhaB1/2CoTdhaT.fwd and dhaB1/2CoTdhaT.rev as primers.

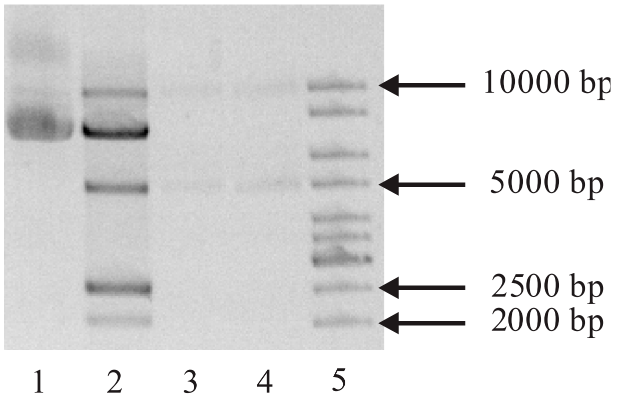

Figure S2. Gel electrophoresis. Lane 1: undigested pMTL83251_$P_{pta\text{-}ack}$_1,3-propanediol_CLOBI. Lane 2: control digestion of pMTL83251_$P_{pta\text{-}ack}$_1,3-propanediol_CLOBI using FastDigest® *Bsp119*I, *Nhe*I e *Xba*I (Thermo Fisher Scientific). Lanes 3 and 4: product of the cloning reaction with pMTL83251_$P_{pta\text{-}ack}$ and the 1,3-PDO gene cluster. Lane 5: GeneRuler DNA Ladder Mix (Thermo Fisher Scientific).

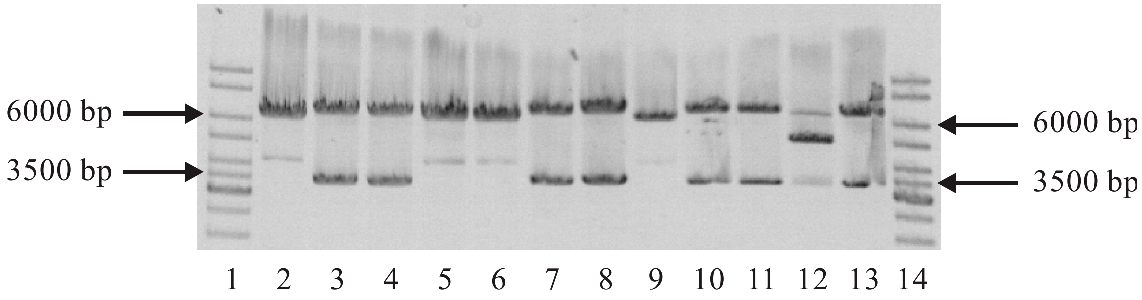

Figure S3. Gel electrophoresis of control digestion with *Pvu*II and *Xba*I. Lanes 1 and 14: GeneRuler DNA Ladder Mix (Thermo Fisher Scientific). Lanes 2 to 13: control digestion of plasmid DNA extracted from a culture of 12 different colonies of *E. coli* XL1-Blue MRF', transformed with pMTL83251_$P_{pta\text{-}ack}$_1,3-PDO_cluster.

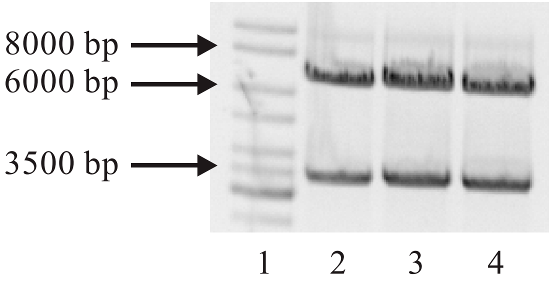

Figure S4. Control digestion of pMTL83251_P$_{pta\text{-}ack}$_1,3-PDO_cluster, extracted from *C. beijerinckii* Br21, using *Pvu*II and *Xba*I. Lane 1: GeneRuler DNA Ladder Mix (Thermo Fisher Scientific). Lanes 2 to 4: plasmid DNA extracted using cultures derived from 3 different colonies of *C. beijerinckii* Br21 [pMTL83251_P$_{pta\text{-}ack}$_1,3-PDO_cluster].
